# A Proposal to Unify the Definition of the Metabolic Syndrome in Children and Adolescents

**DOI:** 10.3389/fendo.2022.925976

**Published:** 2022-06-29

**Authors:** Xin’nan Zong, Pascal Bovet, Bo Xi

**Affiliations:** ^1^Department of Growth and Development, Capital Institute of Pediatrics, Beijing, China; ^2^Center for Primary Care and Public Health, University of Lausanne, Lausanne, Switzerland; ^3^Department of Epidemiology, School of Public Health, Shandong University, Jinan, China

**Keywords:** metabolic syndrome, obesity, blood pressure, hypertension, waist circumference, abdominal obesity, children, adolescents

## Introduction

There was no international consensus how to define the metabolic syndrome (MetS) in children and adolescents so far (1, 2). According to the most widely used MetS definitions in children and adolescents (3, 4), MetS can be diagnosed with abdominal obesity and the presence of two or more other clinical features (i.e., high triglycerides (TG), low high-density lipoprotein cholesterol (HDL-C), elevated blood pressure (BP), or impaired fasting plasma glucose). A recent review clearly pointed out that a key limitation in the area of MetS research was the different definitions and cut-offs of the components of pediatric MetS (5). Static cut-offs for high TG, low HDL-C and impaired fasting plasma glucose which do not depend of sex and age, are widely accepted and used in most of the MetS definitions (3, 4, 6–8). In view of more strong association with sex and age in children and adolescents, sex- and age-dependent percentile cut-offs for defining abdominal obesity and elevated BP are recommended (3, 4, 6, 7, 9, 10), and yet only percentile cut-offs were available at the national level in the past. Due to the lack of unified international cut-offs for abdominal obesity and elevated BP in pediatric population, it is very difficult to make robust global estimates and direct international comparisons of MetS prevalence in children and adolescents across countries/regions worldwide. Therefore, we need to do better to define abdominal obesity and elevated BP in children and adolescents so that we can provide a unified international definition of MetS in children and adolescents for more powerful monitoring and assessment and more coordinated action for global prevention and control of cardiovascular disease.

## What’s Known on the Definitions of Abdominal Obesity and Elevated BP in Children and Adolescents?

The International Diabetes Federation (IDF) and some other groups/researches have recommended to use the 90^th^ percentile of waist circumference (WC) to define abdominal obesity in children and adolescents (3, 4, 7, 9, 10). Similarly, the 90^th^ percentile of BP are also recommended to define elevated BP in children and adolescents (3, 6, 7, 9, 10). However, the 90^th^ percentiles of WC and BP used in these MetS definitions were country-specific WC and BP reference values across different national samples, which was disadvantageous to international comparison of population estimation of abdominal obesity, elevated BP and MetS across different countries/regions. It would therefore be useful to rely on the international 90^th^ percentile cut-offs of WC and BP for the definition of abdominal obesity, elevated BP and MetS in children and adolescents.

## Establishment and Application of International WC Reference Values for Children and Adolescents

In 2020, the international sex- and age-specific WC percentile references were established for children and adolescents between 6 and 18 years based on 72,841 normal-weight children (excluding individuals who were overweight, obese or underweight from the analysis) from eight countries (Bulgaria, China, Iran, Korea, Malaysia, Poland, Seychelles and Switzerland) (11). These international sex- and age-specific 90^th^ percentile WC cut-offs performed well for predicting cardiovascular risk. These international WC cut-offs allowed direct comparison of prevalence of pediatric abdominal obesity across countries/regions. A systematic review stated that these international WC cut-offs have been universally accepted to help identify groups and/or individuals at risk of poor health (12). In 2021, these international WC cut-offs have been recommended by an international expert consensus on pediatric metabolic (dysfunction)-associated fatty liver disease (13).

## Establishment and Application of International BP Reference Values for Children and Adolescents

In 2016, the international sex-, age-, and height-specific BP percentile references (including systolic BP and diastolic BP) were established for children and adolescents between 6 and 17 years based on 52,636 non-overweight children (excluded individuals who were overweight or obese from the analysis) from seven countries (China, India, Iran, Korea, Poland, Tunisia and USA) (14). These international BP references generated from multiple countries were considered as a useful tool for international comparison of elevated BP prevalence in children and adolescents and may help identify hypertensive youths in diverse populations (15, 16). In 2017, these international BP percentiles were recommended as appropriate pediatric normative data by TRUE Consortium (17). In 2019, a scientific statement on cardiovascular risk reduction in high-risk pediatric patients from the American Heart Association stated that these cross-cultural, international BP references could be of additional value in the future (18).

## Proposal and Discussion

We propose that these international 90^th^ percentile cut-offs for WC and BP (11, 14) may be used for the diagnosis of MetS in children and adolescents. We suggest that MetS can be diagnosed among children and adolescents between 6 and 17 years by ①WC≥90^th^ percentile (sex- and age-specific, international WC references (11)) and the presence of two or more other clinical features (i.e., ②TG≥130 mg/dL for 10-17-year-old or ≥100 mg/dL for 6-9-year-old (19), ③HDL-C<40 mg/dL (19), ④systolic BP≥90^th^ percentile or diastolic BP≥90^th^ percentile (sex-, age- and height-specific, international BP references (14)), or ⑤impaired fasting plasma glucose≥100 mg/dL (4, 20)). The greatest value of our proposed unified international MetS definition is to provide a universal framework that allows direct comparison or data synthesis of the MetS prevalence across countries/regions worldwide. Our proposed universal MetS definition is a supplement to the existing definitions and is not intended to replace the existing definitions. Further studies should be conducted to evaluate the short-term and long-term outcomes of those vulnerable individuals with the MetS identified by our proposed universal international definition.

The following points need to be attended. First, younger children, such as the age of 2 to 5 years may also have the potential risk of MetS. Early diagnosis of the MetS at this age was also regarded as an urgent and necessary task (21). A recent prospective cohort study showed risk factor level even in early childhood also linked to adult cardiovascular clinical events (22). We look forward to more evidence and debate on whether the MetS should be diagnosed at this age. Second, like the most widely used MetS definitions in children and adolescents (3, 4), our proposed unified international MetS definition also recommended WC as a proxy of abdominal obesity. WC was considered a good predictor of visceral adipose tissue and an effective measure of abdominal obesity in children and adolescents (23, 24). Many studies have revealed that WC can well predict cardiovascular risk in children and adolescents (25–29), however, a few studies have observed that WC even other anthropometric indices may be not satisfactory markers of metabolic comorbidity (30, 31). We hope that more studies will continue to focus on the association between anthropometric indices and cardiovascular disease in the future. Third, for more accurate evaluation, sex- and age-specific TG and HDL-C values and fasting plasma glucose values should be also considered in the MetS definition in children and adolescents although lipids and glucose level in children and adolescent vary relatively moderately with age. The Lipid Research Clinics Prevalence Study data and the IDEFICS data from large-scale samples could be considered to be used (10, 32) before the emergence of new international data. Fourth, an accurate measurement for WC, systolic and diastolic BP, TG, HDL-C, and fasting plasma glucose must be made following a standardized measurement method so as to present a true MetS estimation and make valid comparison between nations/regions. It should be noted that different lipid measurement methods may have an impact on the results, according to the criteria of the CDC’s Lipid Standardization Program, lipid measurements were recommended for TG using the mass spectrometry–based method and for HDL-C using the ultracentrifugation method (33).

There is no denying that there is controversy and confusion for the MetS concept, but the MetS as an entity is still important and useful, because it identifies a common multiple cardiovascular-risk phenotype that confers a greater risk of cardiovascular disease than a single risk factor (19, 34). Owing to the absence of defined etiology and the unclear implications for clinical care, some pediatric experts preferred focusing attention on the importance of screening for and treating the individual risk factor components of MetS and those children with cardiometabolic risk factor clustering (35). We believe that whether the interest is the MetS or the single risk factor component, the common goal is to reduce the risk and burden of cardiovascular disease and type 2 diabetes.

## Conclusion

Our proposed unified international MetS definition may be useful for population estimation and direct comparison of the MetS prevalence in children and adolescents worldwide, and also for clinical purposes, particularly in countries that have not developed their own national normative data on WC and BP in children and adolescents.

## Author Contributions

XZ: study designed, literature searched and wrote the original draft. PB: study designed and manuscript revised. BX: study designed and manuscript revised. All authors contributed to the article and approved the submitted version.

## Conflict of Interest

The authors declare that the research was conducted in the absence of any commercial or financial relationships that could be construed as a potential conflict of interest.

## Publisher’s Note

All claims expressed in this article are solely those of the authors and do not necessarily represent those of their affiliated organizations, or those of the publisher, the editors and the reviewers. Any product that may be evaluated in this article, or claim that may be made by its manufacturer, is not guaranteed or endorsed by the publisher.
